# Phytomedicine from Middle Eastern Countries: An Alternative Remedy to Modern Medicine against *Candida* spp Infection

**DOI:** 10.1155/2021/6694876

**Published:** 2021-07-14

**Authors:** Mohammad Zubair Alam, Mohd Sajjad Ahmad Khan

**Affiliations:** ^1^Pre-Clinical Research Unit, King Fahad Medical Research Center, King Abdulaziz University, Jeddah 21589, Saudi Arabia; ^2^Department of Medical Laboratory Technology, Faculty of Applied Medical Sciences, King Abdulaziz University, Jeddah 21589, Saudi Arabia; ^3^Department of Basic Sciences, Deanship of Preparatory Year and Supporting Studies, Imam Abdulrahman Bin Faisal University, P.O. Box 1982, Dammam 34212, Saudi Arabia

## Abstract

*Candida* spp are capable of infecting both normal and immunocompromised individuals. More recently, *Candida* infections have spread considerably in healthcare settings, especially in intensive care units, where it is the most frequently encountered pathogen. *Candida albicans* is the commonest species encountered, although infections by non-*albicans* species have also risen in the past few years. The pathogenicity of *Candida* is credited to its aptitude to change between yeast and hyphal modes of growth. *Candida* spp produce biofilms on synthetic materials that protect them and facilitate drug resistance and act as a source for chronic and recurrent infections. Primarily, azoles antifungal agents are utilized to treat *Candida* infection that targets the ergosterol synthesis pathway in the cell wall. The development of antifungal resistance in *Candida* species is a major reason for treatment failure, and hence, there is a need to develop newer antifungal molecules and/or modifications of existing antifungals to make them more effective and less toxic. This has led researchers to oversee the plants to discover newer antimicrobials. Middle Eastern countries are well known for their landscape ranging from dry and sandy deserts to snow-capped mountains. However, they comprise enormous plant diversity with over 20,000 different species showing various types of bioactivities, such as anticancer, antidiabetic, and antimicrobial activities. Especially, the antifungal potential of these phytoproducts could be exploited in the clinical setting for therapy. The present review examines some of the promising alternative natural compounds that have been tested and found effective in treating *Candida* infections *in vitro* in some Middle Eastern countries.

## 1. Introduction

*Candida* spp are ubiquitous and the most common human fungal pathogens that attack both immunosuppressed and immunocompetent individuals and is considered a major healthcare-related infection [1]. In the last few decades, candidemia has been increased radically in healthcare settings and has become the commonest infection encountered in intensive care units [2, 3]. The development mode of *Candida* is mostly unicellular. Although more than 200 species of *Candida* have been identified, only a few species are infectious to humans. *Candida albicans* remains the main species that have been implicated in most of the *Candida* infections, although infections by nonalbicans species have also risen in the past few years [2]. *C. albicans* could reproduce by budding as well as by hyphal form that fragments intermittently to form new mycelia or yeast-like forms. *C. albicans* infection is referred to as candidiasis, which is identified by mucosal infections of gastrointestinal epithelial cells, oropharyngeal mucosa, and vagina. Although *C. albicans* is the foremost infectious agent accountable for candidiasis, nonalbicans species of *Candida* such as *Candida glabrata*, *Candida krusei*, *Candida dubliniensis*, *Candida parapsilosis*, and *Candida tropicalis* have also gained weight due to their regular recovery from infected individuals.

Despite advancements in antimicrobial drug development, it has been expected that approximately 10 million deaths will be caused by drug-resistant pathogens by 2050, which will be greater than the figure of mortalities instigated by cancer [4]. Therefore, it is of the utmost necessity to look for an alternative remedy, such as natural products including plant extracts or oils. It is well-documented that plant extracts/essential oils have a variety of bioactive compounds showing activities such as antimicrobial, anticancer, and anti-inflammatory agents [5, 6]. The antifungal nature of plant products is established by the presence of flavonoids, phenols, saponins, tannins, and terpenoids [7], and the extracts of plants from these regions have been recorded to contain high amounts of these compounds as we have described here. The mechanism of antifungal activity of such molecules is to inhibit cell membrane/wall and progression of the hyphae of the fungus [8]. Moreover, environmental factors such as temperature and availability of water have an impact on the composition of plant extracts/oils [9]. Therefore, it is believed that plants cultivated in the Middle Eastern region, being able to endure severe climate conditions, could offer a range of distinct compounds with a greater variation in bioactivities.

Plants from this region have not been given much importance for exploration compared to their counterparts in other regions. For example, the date palm tree from the African region has been widely investigated for its antibacterial potential and mechanism against *Enterobacteriaceae*, whereas date palm trees from the Middle East have so far less commonly explored for antibacterial action against other *Enterobacteriaceae* [10]. The extreme weather in the Middle Eastern region poses restrictions to extract bioactive compounds from those plants. Therefore, it is crucial to maintain investigation in this region's plants by applying traditional medicine as a reference to surmounting the challenges in modern medicine, leading to the isolation and development of newer and more potent antimicrobial compounds. It is anticipated that information from this review will empower an appraisal of the unequivocal role of Middle Eastern plants in delivering beneficial options to address the difficulties in clinical therapeutics.

## 2. Therapies and Problems Associated with the *Candida* Infections

Azoles are a class of antifungal agents that are most used against *Candida* infections. Based on the number of nitrogen present in the five-membered azole ring, these drugs have two main groups, namely, imidazole (two nitrogen atoms) or triazoles (three nitrogen atoms). Azoles are not fungicidal but fungistatic against filamentous fungi and yeasts that attack the ergosterol synthesis pathway in the cell wall. Ergosterol maintains the fluidity and integrity of the cell wall; hence, inhibition of ergosterol synthesis results in inhibition of fungal growth [11, 12]. Some of the common azole drugs such as fluconazole, itraconazole, posaconazole, and voriconazole inhibit the lanosterol 14-*α*-demethylase encoded by the gene *ERG11*. As a result, the level of ergosterol needed for maintaining a normal cell membrane gets reduced [13]. The lanosterol, 4,14-dimethylzymosterol, and 24-methylene dihydrolanosterol, which are the precursors of ergosterol, are accrued inside the cell and incorporated into the plasma membrane causing alteration in the membrane structure and function. The overall effect of such an accumulation is more drug uptake and water penetration into the cell [14]. Other antifungals commonly used to treat infections are polyenes (amphotericin B and nystatin), echinocandins (caspofungins), allylamines (terbinafine and naftifine), 5-fluorocytosine, and a DNA analog [15]. Polyenes disrupt the structure of the cell membrane through binding to ergosterol causing leakage of intracellular components such as potassium, magnesium, and sugars that consequently leads to the death of the fungal cells [16]. 5-Fluorocytosine is an analog of pyrimidine, enters the cell through cytosine permease, and inhibits thymidylate synthetase meddling with DNA [17]. Caspofungin, micafungin, and anidulafungin belong to antifungals called echinocandins that prevent glucan synthesis in the fungal cell membrane by inhibiting 1,3-*β*-d-glucan synthase that results in weak cell wall and unable to resist osmotic pressure [18]. Allylamines (naftifine and terbinafine) and thiocarbamates inhibit the enzyme squalene epoxidase, which is engaged in the synthesis of ergosterol [19].

Irrespective of the incessant and progressive attainments in the medical field, there are imminent risks that cannot be ignored. One such warning is the upsurge in antimicrobial resistance among pathogens due to overprescription and the promotion of resistance among cancer-curing drugs all the way through DNA mutation in cancerous cells [20]. Moreover, *Candida* species produce biofilms on synthetic materials [21]. Biofilms offer a protected niche for *Candida*, accelerate drug resistance, and act out for chronic infections [22].

The development of antifungal resistance in *Candida* species is a major problem and reason for treatment failure in clinical settings, and hence, there is a need to develop newer antifungal molecules and/or modifications of existing antifungals to make them more effective and less toxic. These newer antifungals can be designed or extracted from plants, animals, or other fungi. The present review examines some of the promising alternative natural compounds that have been tested and found effective in treating *Candida* infections *in vitro* in some Middle Eastern countries. Due to inadequate reviews centering on bioactivities from plant products from the Middle East, we intend to give a discussion on plants from this region that have a variety of bioactivities and to make available evidence on the compounds that can be discovered from these plants. This is to augment our knowledge, improve modern medicine problems such as drug resistance, and achieve an alternative cure for fungal infections.

## 3. Bioactivity of Plant/Extracts from the Middle East

The Middle East is recognized as the most dried-out region on the planet and comprises Bahrain, Cyprus, Egypt, Iran, Iraq, Israel, Jordan, Kuwait, Lebanon, Oman, Palestine, Qatar, Saudi Arabia, Syria, Turkey, the United Arab Emirates, and Yemen. This part of the world has got varied physical geographies, ranging from enormous gravel and sandy deserts to highland plateaux and mountain ranges. Additionally, the climate varies depending on the season; in summer, the temperature lies typically at 38–42°C, whereas during winter, the temperature may fall to 14°C [23].

**Figure 1:**
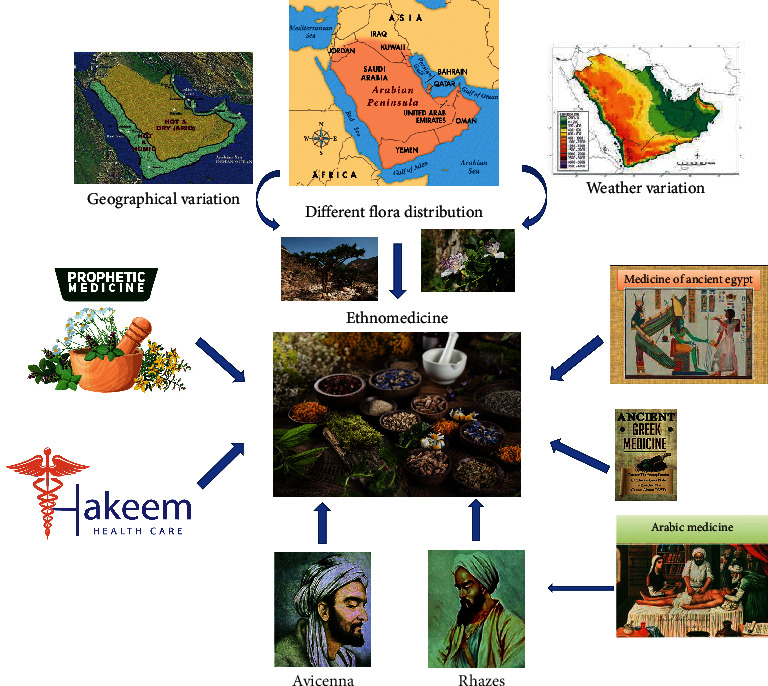
Significance of ethnomedicinal use of plants from the Middle East and Arabian Peninsula (adapted from Aati et al. [24]).

Despite being the driest region in the world, it inhabits over 13,500 species of plants. The most common genera are *Acantholimon*, *Acanthophyllum*, *Astragalus*, *Centaurea*, *Cousinia*, *Dionysia*, *Nepeta*, *Phlomis*, *Salvia*, *Saponaria*, *Silene*, *Stachys*, *Thymus*, and *Verbascum* [20, 23]. The native plants of this region are employed in traditional medicine practiced here, signifying the competence of the Middle Eastern plants to cure several diseases. Ethnomedicinal values of these regional plants are reported since ancient times such as medicines of the Egyptians (3000 BC; pharaohs), the Greeks (400 BC; Hippocrates), and the Romans (37 BC; Dioscorides) [24]. The constant therapeutic use of plants of the Arab peninsula was executed by Prophet Mohammad (peace be upon him, 571–632 AD); a tradition was established as The Prophetic Medicine (*Al-Ṭibb al-Nabawi*) by Ibn Qayyim al-Jawziyya. This practice is still being used in folk medicine in the Arabian world [25]. Historically, this region had presented diverse schools, including the Rhazes; Persian physicians named Abu Bakr Muhammad ibn Zakaria Razi (865–925 AD) and Avicenna; and a Persian physician-philosopher named Ibn Sina (980–1037 AD) and their encyclopedias on ethnomedicine such as *The Law in Medicine* (*Al-Qanun Fi Al-Tibb*) [26], and until now, is contributing to the advancement of herbal medicine. Research has proven that the plants from this region viz. black seeds, costus, fenugreek, garlic, ginger, henna, meswak, and pomegranate are useful in handling human illnesses. Such plant-based medicines are of low cost and have no/lesser side effects [24]. The significance of ethnomedicinal values from this region has been depicted in Figure 1. Here, we have summarized the medicinal plants/extracts reported, from this region for potential anti-*Candida* efficacy. In Table 1, we have listed various plants from this region for their antimicrobial efficacy, which can be exploited to combat the problem of drug resistance to antimicrobial agents.

### 3.1. Saudi Arabia/UAE

In a study, essential oils were extracted from seven different aromatic plants commonly found in the Asir region of southwestern Saudi Arabia and were subjected to antimicrobial and antifungal testing against various bacterial species and *C. albicans*. The plants analyzed for antibacterial activities were *Mentha cervina*, *Ocimum basilicum*, *Mentha pulegium*, *Origanum vulgare*, *Salvia officinalis*, *Ruta graveolens*, and *Scirpoides holoschoenus*. The GC-MS analysis of essential oils from these plant extracts revealed the presence of various bioactive compounds. Some of the major bioactive compounds were pulegone, L-linalool, 1-terpineol, 1-menthone, and eucalyptol detected in the *M. cervina* oil. The major components in *O. basilicum* essential oil were L-linalool (60.9%) and estragole (21.5%). In addition to these major compounds, other compounds were detected in lower amount such pulegone (4.2%), eucalyptol (2.2%), trans-*α*-bergamotene (1.5%), and so on. On the other hand, the major components detected in *O. vulgare* oil were 1-terpineol, sabinene, *γ*-terpinene, *α*-humulene, and *α*-phellandrene [27]. The essential oils of *M. cervina*, *O. basilicum*, and *O. vulgare* were found highly inhibitory against the tested microorganisms in terms of the size of their inhibition zones. The authors of the study reported that essential oils from *M. cervina*, *O. basilicum*, *M. pulegium*, *O. vulgare*, and *R. graveolens* were highly inhibitory against *C. albicans* as the zone of inhibition was more than 30 mm; however, *S. officinalis* and *S. holoschoenus* could not inhibit the growth of *C. albicans*. In terms of minimum inhibitory concentration (MIC) and minimum fungicidal concentration (MFC) as determined through the microdilution method, *M. cervina* essential oil was most effective against *C. albicans* with MIC and MFC values of 0.4 mg/ml and 0.8 mg/ml, respectively [27]. The authors of the study hypothesized that the strong antibacterial activity of these essential oils may be attributed to the presence of the bioactive compounds mentioned above. In terms of the size of the zone of inhibition, the highest activity was observed with *M. cervina* oil. The essential oil of *M. pulegium* exhibited a moderate antimicrobial activity against the strains.

Meswak (*Salvadora persica* L.) is a plant associated with oral hygiene, growing primarily in Saudi Arabia and also in other regions of the Middle East [61]. Roots and stems of meswak plants are reported to be full of silica and resin that can form a shielding layer over an enamel of the teeth, thus safeguarding the teeth from the microbial activity that might lead to the development of caries and gingivitis [62]. In a study by Abubacker et al. [63], the roots and twigs of meswak were extracted with 2% acetic acid, ethyl acetate, 96% ethanol, and water for the evaluation of their activity against oral pathogens *Actinobacillus actinomycetemcomitans* ATCC 43717, *Actinomyces naeslundii*, *C. albicans* ATCC 90028, *Lactobacillus acidophilus* CCUG 5917, *Porphyromonas gingivalis* W50 Black, *Prevotella intermedia* VPI 4197, and *Streptococcus mutans* CCUG 11877. The ethanol extracts of twig and root inhibited the growth of *C. albicans*, and compounds such as N-benzylbenzamide, decane, and stigmasterol were identified to exhibit antimicrobial potential [62].

Soliman and colleagues in Sharjah (UAE) screened *Avicennia marina* (Qurm), *Fagonia indica* (Shoka'a), *Lawsonia inermis* (Henna), *Portulaca oleracea* (Baq'lah), *S. persica* (miwak), *Ziziphus spina-christi* (Sidr), and *Asphodelus tenuifolius* (Kufer) after extraction with ethanol against *C. albicans*. Out of the above-mentioned plants, *L. inermis* and *P. oleracea* were found to exhibit significant anti-*Candida* activity with an MIC of ∼10 *μ*g/ml. Both plant extracts were also able to inhibit *C. albicans* growth at its active growth phases including biofilm development and age resistance. *L. inermis* and *P. oleracea* extracts were also exhibited antibacterial activities against *Staphylococcus aureus*, *Pseudomonas aeruginosa*, *Escherichia coli*, and the multidrug-resistant (MDR) *Acinetobacter baumannii* and *Klebsiella pneumoniae* [28].

Alshubaily synthesized nanoconjugates using chitosan from *Aspergillus niger* and costus extract (NCt/CE). The synthesized NCt, CE, and their nanocomposites were tested against resistant *C. albicans* and *C. glabrata* and were found to exhibit significantly stronger anticandidal activity against the examined strains. NCt/CE nanoconjugates can be used as a novel drug to control resistant pathogenic *Candida* strains [30]. In a study, honey (30, 50, 80, and 95%) and Taif rose oil (1 and 2%) were tested against *Candida* to treat vaginal candidiasis. Four types of monofloral honey were tested such as Markh, Manuka, Qatad, and Sider, and all were inhibitory to the growth of *C. albicans* at 80 and 95% honey concentration. Markh and Manuka honey at 50% concentration completely inhibited the growth of *C. albicans*. Moreover, oil from Taif rose inhibited *C. albicans* completely at 2% concentration following incubation up to 48 hours. The mass spectrometry analysis revealed the presence of gallic acid and quercetin in Markh honey that are known to possess antifungal activity [31].

A common spice, clove (*Syzygium aromaticum*) has been extracted with different organic solvents and tested for activity against *C. albicans*, *C. glabrata*, *and C. tropicalis*. The clove extract was reported to contain eugenol (58.8%), eugenyl acetate (23.8%), trans-Caryophyllene (14.4%), and *α*-Humulene (1.8%). Among all organic extracts of clove, ethyl acetate extract showed the highest inhibitory activity in terms of zone of inhibition against the test species of *Candida* [32]. The meswak (*S. persica*) root stick is widely used throughout the Arabian and Muslim world during ablution before every daily prayer as it is highly recommended for oral hygiene by Prophet Mohammad. The meswak extract (20%) was found highly effective against *C. albicans* and *Enterococcus faecalis* when treated for 6 hours or more, although the same extract was ineffective when treated for 1 hour [33].

The genus *Cyperus* belongs to the family Cyperaceae. There are about 600 species of *Cyperus* distributed worldwide [64]. Many species of genus *Cyperus* have been frequently used as a multi-purpose medicinal plant in folk medicine throughout the world as antidiabetic, anti-inflammatory, antidiarrhoeal, antimutagenic, cytoprotective, apoptotic, antipyretic, analgesic, antioxidant, anticandidal, antimalarial, and antibacterial activities [36, 65, 66]. Al-Hazmi and colleagues found that ethanol and chloroform extract of *C. conglomeratus* exhibited strong anticandidal activity against different *Candida* species. The greatest activity, 3.9 *μ*l, was exhibited by the chloroform and total extract against *C. albicans*. Moreover, the plant extract was highly safe as the LD_50_ was more than 4,000 mg/kg. The toxicity results were further supported by subchronic toxicity test in rats where it was found that no alteration in the liver and kidney functions after administering the rats with 1,000 mg/kg for 15 consecutive days [36]. *Haplophyllum tuberculatum* is a widely used plant for medicinal purposes in the Middle East, including Saudi Arabia. Hamdi and coworkers screened *H. tuberculatum* essentials oils and their compounds for antifungal activity against *C. albicans* ATCC 90028, *C. glabrata* ATCC 90030, *C. parapsilosis* ATCC 27853, and *C. krusei* ATCC 6258. The anti-*Candida* test showed that essential oils from leaves, stems, and their combination strongly inhibited the growth of *C. krusei* at 30 *μ*g/ml essential oil from leaves. Other *Candida* species were moderately inhibited by the essential oils from leaves and stems and their combinations [37]. The active fraction from ethyl acetate extract of *Citrullus colocynthis* was analyzed for its purity by thin-layer chromatography and HPLC. The isolated molecule was further characterized by IR (infrared spectroscopy), ^1^H NMR (nuclear magnetic resonance), ^13^C NMR, and mass spectral analysis. The isolation leads to the novel compound called isopimpinellin with the molecular formula C_13_H_10_O_5_. The compound was found to possess encouraging antimicrobial activity in terms of MIC against *Bacillus subtilis* (100 *μ*g/ml) and *Klebsiella pneumoniae* (75 *μ*g/ml), *Aspergillus niger* (150 *μ*g/ml), and *Candida albicans* (75 *μ*g/ml). Furthermore, the novel compound (isopimpinellin) was found to inhibit fungal biomass. Moreover, isopimpinellin exhibited antibiofilm activity against the uropathogenic strains [38].

The perennial shrub *Deverra tortuosa* is widespread in Al Widyan region, particularly in the valley of Arar, Saudi Arabia [34]. The *D. tortuosa* plant is eaten by various herbivorous animals such as camels. It has been used in traditional medicine for the treatment of constipation, hypertension, and bites [67]. The major component of *D. tortuosa* essential oil is Apiol (65.7 to 74.4%) and has good antioxidant activity. Its essential oil exhibited strong activity against yeasts *Malassezia* spp and *Candida krusei* with MIC values ranging between 2.8 mg/ml and 27 mg/ml [34]. The surface extract of *Psiadia punctulata* collected from Saudi Arabia was investigated for its antimicrobial activity against *S. aureus* and *C. albicans*. The extract was reported to possess antimicrobial activity against *S. aureus* with an MIC of 180 *μ*g/ml and activity against *C. albicans* with an MIC value of 130 *μ*g/ml. Furthermore, the isolated 3′,4′,5,7-tetramethoxyflavone was found to inhibit biofilm formation of *S. aureus* and *C. albicans* by 50 and 90%, respectively, at 40 *μ*g/ml concentration [35].

### 3.2. Turkey

The Turkish flora has been estimated to contain 11,000 taxa out of which 1,280 have been in use as traditional medicines [68, 69]. A study was carried out in Turkey to analyze the phenolic contents of 19 propolis samples using high-performance thin-layer chromatographic (HPTLC). Analysis of different propolis species has shown that O-type was the primarily available propolis in Turkey. Furthermore, researchers have reported a new type of propolis for the first time that was rich in 3-O-methylquercetin (3MQ). It was observed by the researchers of the study that 3MQ-type propolis differs from the O-type. Antimicrobial activities of propolis samples against *S. aureus* (ATCC 6538), *P. aeruginosa* (ATCC 15442), *E. coli* (ATCC 11229), and *C. albicans* ATCC 10231 were determined by disc diffusion and broth dilution methods and found to exert a moderate antimicrobial effect on the tested microorganisms with MIC values ranges between 128 and 512 *μ*g/ml [39].

Some common folk medicinal plants of Turkey such as *Mentha longifolia*, *Mentha piperita*, *Prangos ferulacea*, *Galium verum*, *Salvia limbata*, *Artemisia austriaca*, *Plantago lanceolate*, and *Urtica dioica* were extracted with methanol and chloroform and subjected to evaluation for their anti-*Candida* activities. In total, 102 *Candida* species were tested, of which, 99 were human-pathogenic isolates (35 *C. albicans*, 33. *C. tropicalis*, and 31 *C. glabrata*). Three standard strains were also used in the study as reference. Plants extracted with chloroform did not show any inhibitory effect against any of the *Candida* isolates; however, methanol extracts of these plants were reported to possess strong anti-*Candida* activity [40].

Many species of the genus *Centaurea* have been widely used in traditional medicine in Turkey for various diseases such as dandruff, diarrhea, inflammation, digestion, fever, and infections [41]. Chemical analysis of *Centaurea baseri* essential oil and crude extract showed the presence of hexadecanoic acid (42.3%), nonacosane (8.2%), and heptacosane (8.0%) that were the main components of its essential oil. The extract and essential oil of *C. baseri* were found to exhibit a strong inhibitory effect against *Candida utilis* and *Bacillus cereus* with MIC values of 60 and 47 *μ*g/ml, respectively. The extract of *C. baseri* was also reported to have highly selective cytotoxic properties against various cell lines such as MCF-7, PANC-1, A549, and C6 glioma cells [41].

In a study, *Anthemis stiparum* that is commonly used as a medicinal herb for various ailments was analyzed for its chemical composition, phenolic and flavonoid contents, and antimicrobial and antibiofilm activities. Chemical analysis of its essential oil revealed the presence of germacrene D (11.13%), t-cadinol (11.01%), camphor (6.73%), spathulenol (6.50%), and isoamyl salicylate (6.45%). The methanolic extract constituted 13.6% and 5.9% of pyrocatechol and quercetin, respectively. The methanolic extract was reported with better antimicrobial activity than the essential oil of *A. stiparum* against *S. aureus* (ATCC® 25923) and *Bacillus subtilis* (ATCC® 6633), with an MIC of 1.56 mg/ml. The methanolic extract was found to inhibit *Candida albicans* (ATCC® 10239) adhesion by 80% at 6.25 mg/ml concentration [42].

In another study, several plant species used as traditional medicine in Sakarya province (northwest Turkey) were studied for their antimicrobial activities following extraction with petroleum ether and ethanol. The authors of the study reported that petroleum ether extracts of plants such as *Arum maculatum*, *Datura stramonium*, *Geranium asphodeloides*, and *Equisetum telmateia* were highly inhibitory against *S. epidermidis*, *E. coli*, and *C. albicans* [70].

*Papaver rhoeas* is a red poppy, which is known as “gelincik” in Turkey. The species is found all over Turkey and used for various medicinal purposes such as cough syrup for children and tea for insomnia, sedative, and pain relief [71]. Coban and his team of researchers had collected *P. rhoeas* samples from different parts of Turkey and examined for their alkaloid content as well as their antimicrobial activities against *S. aureus* and *C. albicans*. The samples were reported to contain 12 different types of alkaloids belonging to proaporphine, aporphine, promorphinan, protopine, and rhoeadine groups. The most significant antimicrobial activity was observed with the alkaloid aporphine (roemerine) against *S. aureus* and *C. albicans* with MIC values as low as 1.22 *μ*g/ml and 2.4 *μ*g/ml, respectively [43].

### 3.3. Egypt

A total of 2,174 plant species have been recorded in Egypt; 121 of them are known for their use for medicinal purposes [72]. Historically, Egyptian people in ancient times used to record plants and drugs derived from them on the walls of temples and in the papyri, for example, the famous *Ebers Papyrus*, written in 1550 BC with 876 prescriptions made of 328 different ingredients derived from many plant species. Notable medicinal plant species still in use since ancient civilizations of Egypt are *Artemisia absinthium*, *Acacia nilotica*, *Balanites aegyptiaca*, *Bryonia* sp, *Hyoscyamus muticus*, *Myrtus communis*, *Onopordon* sp, aloe, gums, myrrh, pomegranate, colocynth, linseed, cumin, and *Ziziphus* sp [73].

The plant *Clerodendrum* belongs to the family Lamiaceae and is found in many countries including Egypt. This plant has been reported to be used in treating various diseases and ailments [74, 75]. The leaves of *Clerodendrum chinense* from Egypt were reported to contain phenylpropanoid glycosides (verbascoside, isoverbascoside, and decaffeoylverbascoside), flavonoid (hispidulin), cyclohexylethanoids (cornoside and rengyolone), and icariside B5 [76]. Several species of the genus *Clerodendrum* have been shown to exhibit strong antimicrobial activity [44]. Chloroform extracts of the flowers and stems of *C. chinense* and *C. splendens* were highly inhibitory against *Plasmodium falciparum* and *Trypanosoma cruzi*; however, the same extracts were found moderately effective against *C. albicans* [44].

Ibrahim and coworkers have extracted essential oils from aerial parts of the plants *Calocedrus decurrens*, *Cupressus sempervirens*, and *Tetraclinis articulata*. The extracted essential oils were subjected to antimicrobial activity testing against Gram-positive, Gram-negative bacteria, and *Candida* species. *δ*-3-Carene (43.1%), cedrol (74.0%), and camphor (21.2%) were, respectively, the major components of essential oils of *C. decurrens*, *C. sempervirens*, and *T. articulata*. These essential oils exhibited strong anti-*Candida* activity against several species of *Candida* such as *C. albicans*, *C. glabrata*, *C. krusei*, and *C. parapsilosis* [45].

An antimicrobial study was conducted using formulations prepared with extracts (15%) of henna, pomegranate, and myrrh as well as their blends, and the activity was compared with the marketed gentamycin ointment. The extract formulations were found to exhibit strong antimicrobial activity against *C. albicans*, *S. aureus*, and *E. coli*. The antimicrobial activities of all four formulations (henna, pomegranate, myrrh, and blend) against *C. albicans* were comparable to the commercially available gentamycin ointment. On the other hand, the formulations performed better against bacterial species compared to the gentamycin ointment [46]. The aqueous and organic extracts of *Ceratophyllum demersum*, *Eichhornia crassipes*, *Potamogeton crispus*, and *Potamogeton pectinatus* were tested against *C. albicans* and *C. tropicalis*. The aqueous extract of *P. crispus* was reported to be the most inhibitory against both *Candida* species, followed by the aqueous extract of *E. crassipes*. Although chloroform extracts were also effective against *Candida* species, the ethanol and methanol extracts were only moderately effective [47].

### 3.4. Yemen

In a study from Yemen, the antimicrobial activities of different basidiomycete species were investigated following extraction with dichloromethane, methanol, and aqueous extracts. The methanol extracts of *Chlorophyllum molybdites*, *Ganoderma xylonoides*, *Trametes cingulate*, *Agaricus bernardii*, *Agrocybe pediades*, *Coriolopsis polyzona*, *Pycnoporus sanguineus*, and *Trametes lactinea* were reported with strong inhibitory effect against bacterial species tested. On the other hand, methanol extracts of *C. molybdites*, *G. xylonoides*, and *P. sanguineus* were found with considerable antifungal activity [48]. The volatile oil obtained from *Ganoderma pfeifferi* (a basidiomycete) was examined for chemical composition and antimicrobial activity. The antimicrobial effects of volatile oil were tested against 5 bacteria and 2 *Candida* species (*C. albicans* and *C. maltosa*) using disc diffusion and broth microdilution methods. Ninety percent of volatile oil corresponded to 4 compounds, namely, amyl-vinyl-carbinol (73.6%) followed by 1-octen-3-ol acetate (12.4%), phenylacetaldehyde (3.0%), and 6-camphenol (1.5%). The volatile oil was more inhibitory to Gram-positive bacteria than to Gram-negative ones. The oil exhibited strong antimicrobial activity against *C. albicans* with an MIC of 600 *µ*g/ml [49].

The essential oil from the leaves of *Stachys yemenensis* was analyzed for its chemical composition and antimicrobial activity. The essential oil consisted was *α*-phellandrene (13.9%), *β*-phellandrene (11.7%), elemol (12.0%), spathulenol (6.7%), *β*-eudesmol (5.0%), *α*-eudesmol (4.75%), and squalene (4.8%). The essential oil in concentration ranged between 0.001% (v/v) and 2.5% (v/v) was tested by using the broth dilution method against *E. coli* ATCC 35218, *S. aureus* ATCC 43300, and two clinical strains, *C. albicans* and *C. glabrata*. MIC value against *E. coli* strain was 0.15% (v/v) in hydro-distilled extract and 0.3% (v/v) for supercritical fluid extract. For *S. aureus*, the MIC values were 0.6% (v/v) in supercritical fluid extract and 2.5% (v/v) in hydro-distilled. The hydro-distilled and supercritical fluid extract of *S. yemenensis* essential was not strongly inhibitory against the *C. albicans* and *C. glabrata* as MIC values were higher than 2.5% (v/v) except for *C. glabrata*, for which the MIC of the supercritical fluid extract was 2.5% (v/v) [50].

### 3.5. Jordan

Jordan although a small country contains around 2,500 plant species in which 285 species have been described as medicinal plants [77]. Talib and Mahasneh prepared 51 extracts from 14 different plants and using ethanol, methanol, water, butanol, and n-hexane. Of these extracts, 22 were effective against several bacteria with MIC values between 62.5 and 1,000 *μ*g/ml. The butanol extract of *Rosa damascena* inhibited 100% of *Salmonella typhimurium* and *Bacillus cereus* at concentrations of 62.5 and 250 *μ*g/ml, respectively. Furthermore, the butanol extract of *Narcissus tazetta* and aqueous extract of *Rosa damascena* receptacles were highly inhibitory against *C. albicans* at a concentration of 125 *μ*g/ml [51].

One of the most important genera of the family Asteraceae is *Anthemis*, which comprises nearly 210 species. It is distributed across Europe, Southwestern Asia, Northern and Northeastern Africa, Southern Arabia, and tropical East Africa [78]. Various species of *Anthemis* have been in use in traditional medicine since the time of the Roman Empire [79, 80]. Recent studies suggested that several species of *Anthemis* have tremendous antimicrobial potential that could be correlated with their phenolic and flavanoid compositions [81]. *Anthemis palestina* is mostly found in the middle and northern mountainous regions of Jordan and is known as Palestine chamomile due to its resemblance to Roman and German chamomiles [52]. Bardaweel and colleagues characterized the antimicrobial activity among many other activities of *A. palestina*. They extracted oil from *A. palestina* through hydro-distillation and tested against many bacterial and *Candida* species including *C. albicans*, *C. glabrata*, and *C. krusei*. The extracted oil was reported to possess a broad spectrum of antimicrobial activity, especially against Gram-positive bacteria. Furthermore, the oil was also inhibitory for *C. albicans*, *C. glabrata*, and *C. krusei* having MIC values ranging between 48 and 95 *μ*g/ml [52].

*Artemisia judaica* is another plant widely used in Jordanian folk medicine. *A. judaica* has been reported for the treatment of inflammation and infections caused by fungi, bacteria, and viruses [82]. *A. judaica* when extracted with a mixture of water and ether was found to inhibit *Klebsiella aerogenes*, *P. aeruginosa*, and *S. aureus* [53]. It has also been reported to reduce blood glucose levels in diabetic rats [83]. Abu-Darwish and colleagues have reported that the major constituents of *A. judaica* oil were piperitone (30.4%), camphor (16.1%), and ethylcinnamate (11.0%). *Cryptococcus neoformans* was found to be highly susceptible to the oil from *A. judaica* with an MIC value of 0.16 *μ*l/ml. The interesting part of the oil from *A. judaica* was its strong inhibitory effect on germ tube formation in *C. albicans*. Eighty percent inhibition of filamentation was recorded at a concentration of 0.16 *μ*l/ml. Moreover, the oil successfully disrupted the preformed biofilms in terms of reducing the amount of biomass attached [53].

### 3.6. Israel/Palestine

The human oral cavity is a habitat for a large number of microorganisms including various *Candida* species. At least 20 different species are known to inhabit the oral cavities of normal and immunocompromised persons. Adwan and coworkers carried out a study to investigate and compare anti-*Candida* activities of nine different commercial toothpaste with and without herbal extracts against 45 oral and nonoral *Candida* isolates [84]. Although all toothpaste were effective in inhibiting the growth of *Candida* isolates, but the toothpaste containing herbal extracts were found to exhibit higher anticandidal activity [84]. The plant *Scolymus maculatus* (golden thistle) is believed to be curative for many diseases in Palestinian region. Different extracts of *S. maculatus* were investigated for their chemical composition and antimicrobial activity. The extracts were reported to contain stigmasterol, *γ*-sitosterol, lupeol, lupeol acetate, and *β*-amyrin among many others. MICs of *S. maculatus* extracts were determined against *S. aureus*, *S. typhimurium*, and *C. albicans*. Different organic extracts of *S. maculatus* exhibited an MIC of 500 *μ*g/ml against bacterial isolates and *C. albicans*, whereas aqueous extract was having a higher MIC value [54].

Many plants, particularly carnivorous plants, produce naphthoquinones that have been reported for antifungal activities [85]. In a study, two naturally occurring naphthoquinones, namely, droserone and its methylated derivative 5-O-methyldroserone, were isolated from pitchers of *Nepenthes khasiana* (a carnivorous plant). The antifungal activities of droserone and 5-O-methyldroserone were compared. The antifungal activity revealed that droserone was more active when used as an element of the pitcher liquid or as a purified compound. When tested against *Candida* and *Aspergillus* spp., the inhibitory and fungicidal effects were observed at a significantly lower concentration than the cytotoxic amount in the cells of a human embryonic kidney cell line, 293T. These naturally occurring naphthoquinones may lead us to develop newer antifungal drugs with reduced toxicity [55].

Various species of the genus *Lavandula* (lavender) have been in use for a long time for medicinal purposes in Palestine region. Three species are native to the Palestine region, namely, *Lavandula pubescens*, *Lavandula stoechas*, and *Lavandula coronopifolia* [86]. Several pharmacological properties have been reported for lavender oils such as anesthetic, sedative, analgesic, anticonvulsant, antispasmodic [87, 88], antibacterial and antifungal effects, and inhibition of microbial resistance [89]. They are also used for the treatment of inflammation and many neurological disturbances [90]. The essential oil of *L. pubescens* has been reported to possess strong antibacterial activity *in vitro* against many types of bacteria such as *Salmonella enterica*, *S. aureus*, *Micrococcus luteus*, *E. faecalis*, and *E. coli* [89, 91]. Many products derived from Palestinian Downy lavender (*L. pubescens*) have been utilized for centuries as herbal medicine in the region [86]. The phytochemical analysis of essential oil from *L. pubescens* revealed the presence of many compounds in which carvacrol (a type of monoterpene) was reported as the most abundant (65.3%). The essential oil from *L. pubescens* has been shown to demonstrate strong antibacterial activity against *S. aureus* with 95.7% inhibition. The essential oil was also found to exhibit strong inhibitory activity against *C. albicans* with an MIC value of 0.47 *μ*l/ml. Furthermore, the oil was also reported to possess antidermatophyte activity against *Microsporum canis*, *Trichophyton rubrum*, *Trichophyton mentagrophytes*, and *Epidermophyton floccosum* [56].

### 3.7. Iran

The figwort (*Scrophularia* sp) is a commonly found plant in Iran, spread across the mountain ranges and deserts [92]. The phytochemical analysis of stem, rhizome, and seed extracts of this plant has revealed the presence of a higher amount of phenolic compounds, which is credited to its antimicrobial potential [93]. A study conducted by Vahabi et al. [94] revealed the antimicrobial activity of figworts (*S. striata*) against oral pathogens. A systematic review of the medicinal properties of *Dracocephalum kotschyi* and its significance in Iran has highlighted potential health benefits of this plant from this region. The plant has been well-documented for its antioxidant, antitumor, and antimicrobial efficacies against *C. albicans* [95]. Mansourian and colleagues [57] tested resistant *C. albicans* isolates from patients and reported the anti-*Candida* activity of *Syzygium aromaticum* extract as significantly better than the activity of nystatin (*P* < 0.001). *Achillea millefolium* (common name: yarrow), which belongs to the family Asteraceae, is traditionally used for various female genital disorders such as inflammations, infections, and dysmenorrhea [96, 97]. The plant has been reported to possess strong anti-*C. albicans* as well as resistance against other fungal pathogens [98]. Zakeri and coworkers have conducted a study to evaluate the effectiveness of *A. millefolium* (2%) extract in the cream formulation in comparison with clotrimazole vaginal cream in patients with vulvovaginal candidiasis. The study was conducted on 80 women diagnosed with vulvovaginal candidiasis. Half of the women were given clotrimazole 1% vaginal cream, and the remaining women were given vaginal cream containing the aqueous extract of *A. millefolium* for 7 days. The results were suggestive that vaginal cream containing *A. millefolium* could reduce the complaints of vulvovaginal candidiasis [58]. In a study, the essential oil of *D. kotschyi* was analyzed using GC-MS. In cultivated plants, major compounds of essential oil were *α*-pinene (13.66%), (E)-citral (12.89%), neral (11.25%), methyl geranate (8.66%), limonene (8.33%), campholenal (6.22%), and geraniol (5.69%). On the other hand, a stark difference was noted in the composition of EO in naturally grown plants in which two main compounds, cyclohexylallene (52.63%) and limonene (35.88%), were predominant. The antimicrobial activities of this plant were determined against 12 different microorganisms. The MIC value against *Bacillus subtilis* was 31.25 *μ*g/ml; the MIC was lower than that obtained by treating the same strain with Rifampin. The EO obtained from wildly growing *D. kotschyi* was highly inhibitory against *C. albicans* (MIC value of 31.25 *μ*g/ml) lower than that obtained treating the yeast with Nystatin [59]. *Peganum harmala* (wild rue) is used as a topical antifungal agent for treating many infections in Iran. In a study, *P. harmala* was investigated for its inhibition of *C. albicans* biofilm formation. *C. albicans* (27) were collected from women with vaginitis and grown to form biofilm and then *P. harmala* EO was applied as an antibiofilm agent. The researchers noted that *P. harmala* EO in the concentration of 12 *μ*g/ml strongly inhibited biofilm formation. Moreover, at lower concentrations (10 and 6 *μ*g/ml), the essential oil of *P. harmala* was also effective in controlling biofilm formation albeit weakly [60].

Chicory (*Cichorium intybus*) is a plant native to the Iranian region and has been documented to possess antifungal assets [99]. The ethanol extracts of chicory leaves were evaluated against *C. glabrata* and *C. krusei* by Eslami et al. [100]. They observed that *C. krusei* is more sensitive to the chicory ethanol extract compared to *C. glabrata*. They found that the antifungal activity of chicory leaf extract is due to the presence of compounds such as lactucin, lactucopicrin, deoxylactucin, and *α*-1,3-dihydrolactucin. The compounds identified were sesquiterpenes that inhibited the growth of yeast by disrupting the membranous structure of the *Candida* cells [7, 101].

### 3.8. Cyprus

*Cupressus sempervirens* is a medicinal tree mostly found in Mediterranean region, which has been widely used to treat several ailments such as stomach pain, diabetes, inflammation, laryngitis, and contraceptives [102]. Its leaves and seeds have been used to treat wounds, ulcers, bruises, sores, pimples, pustules, and skin eruptions, as well as the essential oil from the leaves and cones is used externally for headache, colds, cough, and bronchitis. Because of its several medical advantages, *C. sempervirens* is used as a cosmetic ingredient in perfumery and soap-making [103]. In a study, the chemical composition of hydro-distilled essential oil of *C. sempervirens* was analyzed by a GC and GC-MS system. The analysis revealed the presence of constituents like *α*-pinene (48.6%), *δ*-3-carene (22.1%), limonene (4.6%), and *α*-terpinolene (4.5%) were the main components comprising 79.8% of the oil. The methanol extract of *C. sempervirens* was highly inhibitory to the growth of the bacteria studied. The essential oil and methanol extract of *C. sempervirens* eradicated the biofilm from the surface and thus can be employed as a natural preservative in food and pharmaceuticals [29].

### 3.9. Other Countries of the Region

In a study, Barbour et al. [104] evaluated the *in vitro* antimicrobial potential of several water and methanol extracts obtained from diverse parts of 27 ethnic wild plants used in Lebanese folk medicine. The microorganisms under study were *Escherichia coli*, *Proteus* sp, *Pseudomonas aeruginosa*, *Shigella dysenteriae*, *Salmonella enteritidis*, *Salmonella typhi*, *Staphylococcus aureus*, *Streptococcus faecalis*, and *Candida albicans*. Out of 39, only 1 water extract from *Alchemilla diademata* whole plant exhibited antimicrobial activity against *Staphylococcus aureus*. However, methanol extracts of 10 other plants were successful by showing susceptibility to test organisms such as *Achillea damascena* whole plant (88.8%), *Anthemis scariosa* flowers (88.8%), *Cirsium* sp whole plant (88.8%), *Centaurea ainetensis* flowers (88.8%), *Hieracium* sp whole plant (88.8%), *Origanum libanoticum* whole plant (99.9%), *Ranunculus myosuroides* whole plant (88.8%), *Nepeta curviflora* leaf (88.8%), *N. curviflora* stem, and *Verbascum leptostychum* flower (99.9%). They concluded that the antimicrobial activities of plants were more evident in methanol than water extracts of the indigenous plants of Lebanon.

In a study from Qatar, aqueous, ethanol, and butanol crude extracts of the aerial parts of ten native plants viz. *Avicennia marina*, *Lotus halophilus*, *Pulicaria gnaphaloides*, *Capparis spinosa*, *Medicago laciniata*, and *Limonium axillare* exhibited varied levels of antimicrobial activities against *E. coli*, *P. aeruginosa*, *B. cereus*, *S. aureus*, *C. albicans*, and *A. flavus* [105]. On the other hand, ten indigenous Bahraini plants were evaluated by Mandeel and Taha [106] against a variety of fungal pathogens. It was observed that ethanol extract of *Cressa cretica* L. was highly effective against *Penicillium citrinum* and *Candida albicans*. Their study also highlighted that diffusible metabolites of *Heliotropium curassavicum* have marked inhibitory effects against these pathogens. However, chloroform extract of *Emex spinosa* exhibited strong activity against *Alternaria alternata* and *Saccharomyces cerevisiae*. However, the highest growth inhibition was recorded for *Fagonia indica* against *Penicillium citrinum*.

Organic and aqueous extracts from fruits, leaves, and roots of *Tribulus terrestris* L., an Iraqi medicinal plant, were studied by Al Bayati and Al Mola [107] for their antimicrobial potential against 11 species of pathogenic and nonpathogenic microorganisms including *Candida albicans*. The extracts from the different parts of the plant were potential antimicrobials against the majority of microorganisms. The ethanol extract from the fruits was the most active against Gram-positive and Gram-negative bacteria and showed the highest antifungal action against *C. albicans* (MIC value of 0.15 mg/ml). Three types of dates from Oman (Mabseeli, Um-sellah, and Shahal) have also been described to possess strong antimicrobial activities [108]. The antimicrobial activities of date seeds are due to the occurrence of high amounts of phenolic compounds, such as p-coumaric, ferulic and sinapic acids, flavonoids, and procyanidins [109].

## 4. Conclusion

In the Middle East region, using plant materials as medicine has a very long history. Many of the current local medicinal plants were found described on clay tablets from the Mesopotamian civilizations such Sumerians, Assyrians, Akkadians, and Hittites [110]. The published literature indicates that plant extracts/oils could be good candidates for addressing the ever-growing problem of drug resistance in bacteria and *Candida* strains. Many researchers have reported promising anti-*Candida* activities of aqueous and organic extracts prepared from different parts of several medicinal plants. In this review, it has been demonstrated that plants from the Middle Eastern region are competent to show potential antimicrobial, especially anti-*Candida* activities. Studies have also reported major bioactive compounds that could be developed as a drug candidate either alone or potentiating the efficacy of existing antifungals by reducing the side effects. We have compiled here the Middle Eastern plant extracts/oils/products as a potential remedy for the challenges encountered in the clinical setting, such as the surge in drug resistance. We hope this work could facilitate more research to explore plants from this region to reveal the precise mechanisms of action. A larger number of studies have been conducted *in vitro*, and only fewer studies have been carried out in humans, so there is a great need and scope for further improvement in studies via clinical trials in treating *Candida* infections.

## Figures and Tables

**Table 1:** Plants or their extracts reported from Middle Eastern countries with anti-*Candida* activities.

Country	Plant or extract	Effects on *Candida* species	Reference
Saudi ArabiaUAE	*Mentha cervina*	EO highly inhibitory; IZ = 43 mm	[27]
	*Ocimum basilicum*	EO highly inhibitory; IZ = 45 mm	
	*Ruta graveolens*	EO highly inhibitory; IZ = 30 mm	
	*Mentha pulegium*	EO highly inhibitory; IZ = 39 mm	
	*Origanum vulgare*	EO highly inhibitory; IZ = 42 mm	
	*Scirpoides holoschoenus*	EO moderately inhibitory; IZ = 17 mm	
	*Salvia officinalis*	EO no effect on growth inhibition	
	*Lawsonia inermis*	EE inhibitory, IZ = 22 mm; % growth inhibition significant; MIC = 10 *μ*g/ml	[28]
	*Portulaca oleracea*	EE inhibitory, IZ = 17 mm; % growth inhibition significant; 10 *μ*g/ml	
	*Salvadora persica*	EE inhibitory, IZ = 17 mm; % growth inhibition significant; 25 *μ*g/ml	
	*Asphodelus tenuifolius*	EE moderately inhibitory, IZ = 11 mm; % growth inhibition significant; 50 *μ*g/ml	
	*Cupressus sempervirens*	EO and ME eradicated *Candida* biofilm from the surface	[29]
	*Aspergillus niger and costus extract*	Nanoconjugates were having strong anti-*Candida* activity	[30]
	Honey (four types Markh, Manuka, Qatad, Sider)	Markh and Manuka honey completely inhibited *Candida albicans* growth at 50% concentration	[31]
	Taif rose oil	Complete inhibition of *C. albicans* at 2% oil	
	*Syzygium aromaticum*	DEE IZ = 19 mm (*C. albicans*); IZ = 16 mm (*Candida glabrata*); IZ = 24 (*Candida tropicalis*)	[32]
		EAE IZ = 21 mm (*C. albicans*); IZ = 15 mm (*C. glabrata*); IZ = 31 mm (*C. tropicalis*)	
		ME IZ = 16 mm (*C. albicans*); IZ = 13 mm (*C. glabrata*); IZ = 25 mm (*C. tropicalis*)	
		HE IZ = 19 mm (*C. albicans*); IZ = 10 mm (*C. glabrata*); IZ = 30 mm (*C. tropicalis*)	
	*Salvadora persica*	EE meswak extract (20%) was highly inhibitory against *C. albicans*	[33]
	*Deverra tortuosa*	EO strongly active against *Malassezia* spp and *Candida krusei*; MIC 2.8 and 27 mg/ml, respectively	[34]
	*Psiadia punctulata*	DCME MIC against *C. albicans* 130 *μ*g/ml	[35]
	3′, 4′, 5, 7-Tetramethoxyflavone	90% of *C. albicans* biofilm inhibition at 40 *μ*g/ml	
	*Cyperus conglomeratus*	Ethanol and chloroform extracts exhibited strong anti-*Candida* activity; the plant extract was safe as the LD_50_ was more than 4,000 mg/kg	[36]
	*Haplophyllum tuberculatum*	EO was active against *C. albicans*, *C. glabrata*, *C. parapsilosis*, and *C. krusei*	[37]
	*Citrullus colocynthis*	Ethyl acetate extract contained isopimpinellin and exhibited antibiofilm activity	[38]

Turkey	Propolis (19 different types)	MIC ranged between 128 and 512 *μ*g/ml against *C. albicans*	[39]
	*Mentha longifolia*, *Mentha piperita*, *Prangos ferulacea*, *Galium verum*, *Salvia limbata*, *Artemisia austriaca*, *Plantago lanceolate*, *Urtica dioica*	Strong anti-*Candida* activity in ME	[40]
	*Centaurea baseri*	EO had strong inhibitory effect against *Candida utilis*; MIC 60 *μ*g/ml	[41]
	*Anthemis stiparum*	ME exhibited better antimicrobial activity than EO; 80% inhibition in adhesion of *C. albicans* at 6.25 mg/ml concentration	[42]
	*Papaver rhoeas*	PEE alkaloid aporphine highly effective against *C. albicans*; MIC 2.4 *μ*g/ml	[43]

Egypt	*Clerodendrum chinense C. splendens*	CHE was highly inhibitory for *Plasmodium falciparum* and *Trypanosoma cruzi*; moderately effective against *C. albicans*	[44]
	*Calocedrus decurrens*, *Cupressus sempervirens*, *Tetraclinis articulata*	EOs from these plants exhibited strong anti-*Candida* activity against *C. albicans*, *C. glabrata*, *C. krusei*, and *C. parapsilosis*	[45]
	Henna, pomegranate, myrrh	15% extracts (individual and mix) were comparable to commercial gentamycin in inhibiting *C. albicans*	[46]
	*Potamogeton crispus*	AE strong activity against *C. albicans*	[47]
	*Eichhornia crassipes*		

Yemen	*Chlorophyllum molybdites*, *Ganoderma xylonoides*, *Pycnoporus sanguineus*	ME exhibited considerable antifungal activities	[48]
	*Ganoderma pfeifferi*	VO was more effective to Gram-positive than Gram-negative bacteria. VO exhibited strong activity against *C. albicans*; MIC 600 *μ*g/ml	[49]

Jordan	*Stachys yemenensis*	EO was moderately effective on *C. glabrata*	[50]
	*Rosa damascene*	AE was highly inhibitory against *C. albicans* at a concentration of 125 *μ*g/ml	[51]
	*Narcissus tazetta*	BE was highly inhibitory against *C. albicans* at a concentration of 125 *μ*g/ml	
	*Anthemis palestina*	EO was inhibitory to *C. albicans*, *C. glabrata*, and *C. krusei*; MICs between 48 and 95 *μ*g/ml	[52]
	*Artemisia judaica*	EO of *Cryptococcus neoformans* was found highly susceptible EO of *A. Judaica*; an MIC of 0.16 *μ*l/ml; strong inhibitory effect on germ tube formation in *C. albicans*; up to 80% inhibition at 0.16 *μ*l/ml; and disrupted preformed biofilm of *C. albicans*	[53]

Israel and Palestine	*Scolymus maculatus*	Various organic extracts exhibited an MIC of 500 *μ*g/ml against *C. albicans*	[54]
	*Nepenthes khasiana*	Naturally occurring naphthoquinones (droserone and 5-O-methyldroserone) were highly effective against *Candida* Spp and *Aspergillus* Spp	[55]
	*Lavendula pubescens*	EO exhibited strong inhibitory activity against *C. albicans* and possessed antidermatophyte activity against *Microsporum canis*, *Trichophyton rubrum*, *Trichophyton mentagrophytes*, and *Epidermophyton floccosum*	[56]

Iran	*Syzygium aromaticum*	Significantly better anti-*Candida* activity than the activity of Nystatin	[57]
	*Achillea millefolium*	Vaginal cream containing *A. millefolium* (2%) reduced the complaints of vulvovaginal candidiasis	[58]
	*Dracocephalum kotschyi*	The EO from wildly growing *D. kotschyi* was more inhibitory against *C. albicans* than Nystatin	[59]
	*Peganum harmala*	Highly effective against *C. albicans* biofilm formation	[60]

*Note.* IZ = inhibition zone; EO = essential oil; AE = aqueous extract; HE = hexane extract; BE = butanol extract; CHE = chloroform extract; ME = methanol extract; EE = ethanol extract; EAE = ethyl acetate extract; DCME = dichloromethane extract; DEE = diethylether extract; PEE = petroleum ether; and VO = volatile oil.

## Data Availability

No data were used to support this study.
